# Ketoacidosis at first presentation of type 1 diabetes mellitus among children: a study from Kuwait

**DOI:** 10.1038/srep27519

**Published:** 2016-06-22

**Authors:** Azza Aly Shaltout, Arshad Mohamed Channanath, Thangavel Alphonse Thanaraj, Dina Omar, Majedah Abdulrasoul, Nabila Zanaty, Maria Almahdi, Hessa Alkandari, Dalia AlAbdulrazzaq, Linda d’Mello, Fawziya Mandani, Ayed Alanezi, Eman AlBasiry, M. Alkhawari

**Affiliations:** 1 Dasman Diabetes Institute, Pediatric Research Unit, Capital 15462 Kuwait; 2 Dasman Diabetes Institute, Department of Integrative Informatics, Capital 15462 Kuwait; 3 Faculty of Medicine, Kuwait University, Jabriya 13110 Kuwait; 4 Adan Hospital, Department of Pediatrics, Ahmadi 52853 Kuwait; 5 Farwaniya Hospital, Department of Pediatrics, Farwaniya 81004 Kuwait; 6 Sabah Hospital, Department of Pediatrics, 90801 Kuwait; 7 Jahra Hospital, Department of Pediatrics, 1753 Jahra, Kuwait; 8 Mubarak Hospital, Department of Pediatrics, 43787, Hawalli, Kuwait; 9 Amiri Hospital, Department of Pediatrics, Capital 13041 Kuwait

## Abstract

We examined the frequency and severity of diabetic ketoacidosis (DKA) in 679 children and adolescents (0–14 years) at diagnosis of Type 1 Diabetes Mellitus (T1DM) in Kuwait. Between 1^st^ January 2011 and 31^st^ December 2013, all newly diagnosed children with diabetes were registered prospectively in a population-based electronic register. DKA was diagnosed using standard criteria based on the levels of venous pH and serum bicarbonate. At the time of diagnosis, mild/moderate DKA was present in 24.8% of the children, while severe DKA was present in 8.8%. Incidence of ketoacidosis was significantly higher in young children less than 2 (60.7% vs 32.4% p = <0.005) compared to children 2–14 years old, and a higher proportion presented with severe DKA (21.4% vs 8.3% p = <0.05). No association was seen with gender. Significant differences were found in the incidence of DKA between Kuwaiti and non-Kuwaiti children (31.1% vs 39.8%; p < 0.05). Family history of diabetes had a protective effect on the occurrence of DKA (OR = 0.44; 95% CI = 0.27–0.71). Incidence of DKA in children at presentation of T1DM remains high at 33.6%. Prevention campaigns are needed to increase public awareness among health care providers, parents and school teachers in Kuwait.

Diabetic ketoacidosis (DKA) is a serious life threatening complication of type 1 diabetes mellitus (T1DM) and constitutes a medical emergency with significant morbidity and mortality^1^, mostly due to cerebral edema during the course of resuscitation^2^,^3^. Worldwide, approximately 65,000 children aged under 15 years develop T1DM each year, and 13% to 80% of these children present with DKA at the time of diagnosis^4^. The highest frequencies for DKA at presentation of T1DM are seen in Saudi Arabia (44.9%)^5^, Taiwan (65%), Romania (67%), and the United Arab Emirates (80%), and the lowest in Hungary (23%), Finland (22%), Canada (18.6%) and Sweden (14%)^4^.

During the period 1990–1999, the multinational World Health Organization (WHO) sponsored Diabetes Mondiale (DiaMond) Project, has reported worldwide variations in incidence of T1DM in children, by means of using standardized incidence data from 57 countries^4^,^6^. Utilizing the DiaMond protocol on data from Kuwait, during the 1990s, a high incidence of T1DM, was reported from Kuwait^7^, rising from 15.4 to 20.9 per 100000^8^, and nearly half of the children aged 0–14 years presented with DKA^9^.

Recently, there has been an increasing global interest to assess the incidence rates of DKA^10^. However, reports on DKA from Arab countries are scarce and are limited to few studies from Kuwait^9^,^11^, Saudi Arabia^5^,^12^,^13^, Oman^14^ and the United Arab Emirates^15^.

In the present study, we report the incidence and severity of DKA in children who presented with T1DM in 2011–2013 in Kuwait, and explore the trend of frequency of DKA over the last two decades.

## Methods

### Setting

Kuwait is divided into six administrative health regions and each of the six regional offices maintains at least one public hospital and a number of primary health care centers and specialized diabetes clinics. Children at diagnosis of diabetes are admitted to specialized pediatric diabetes and endocrine units in general hospitals, for stabilization, management and education by a multidisciplinary team. Medical care is free of charge in Kuwait, and insulin, insulin analogs, pens are provided free at the point of care to all children with diabetes. Data were derived from a nationwide registry for incident cases of T1DM, the Childhood-Onset Diabetes electronic Registry (CODeR). This initiative is part of the Kuwait-Scotland eHealth Innovation Network (KSeHIN), a partnership between the Kuwait Ministry of Health (MoH), the University of Dundee, and Aridhia Informatics Ltd. established in October 2010. The network provides an integrated package of clinical service development, education and research, all underpinned by state-of-the-art technology^16^. Briefly cases are identified by authorized individuals who undertake regular review of case records obtained from both primary and secondary care.

### Data Repository

The repository, maintained at Dasman Diabetes Institute, captures clinical details of children aged 0–14 years and upwards in Kuwait utilizing a unique civil identifier number. The period of study was 2011 to 2013. Population of children aged 0–14 in 2013 was 670351 (348816 males and 321565 females) of which 60% are Kuwaiti nationals^17^. The registry utilizes capture-recapture method to estimate the degree of ascertainment^17^, validating the primary data source, enabling calculation for ascertainment corrected incidence, as recommended by the WHO DiaMond Project^6^. The primary data source was active hospital reporting; the secondary data source was reported by family physicians and general practitioners in primary healthcare centers distributed all over the country, and the Kuwait Diabetes Society. The ascertainment rate in children aged 0–14 years was 96.7%.

### Data collection

Data used in this study was prospectively collected using standardized case report forms which were then directly inputted into CODeR online application (https://coder.health.org.kw). All children (aged 0–14 years) newly diagnosed for T1DM at hospitals in Kuwait were included. Diagnosis was established according to WHO criteria^18^ and the date of onset was taken as the date of first insulin injection. In this study, recommendations endorsed by ISPAD Clinical Practice Consensus Guidelines 2014^19^ were used to define DKA and to classify severity. Severity is defined as mild when pH is <7.3 and/or bicarbonate of <15 mmol/L, as moderate when pH is <7.2 and/or bicarbonate of <10 mmol/L and as severe if pH is <7.1 and/or bicarbonate was <5 mmol/L. Standard definitions (namely that bicarbonate level is >15 mmol/L) were used to denote absence of DKA in children at onset of T1DM. Data included age, gender, nationality, date of birth, date of diagnosis, type of diabetes, clinical characteristics, family history of diabetes in first degree relatives, plasma glucose, glycated haemoglobin (HbA1c), venous pH, blood bicarbonate and urinary ketones. Data on mortality and morbidity due to cerebral edema was collected.

### Testing for different forms of diabetes

Pancreatic autoantibody testing was performed in few centers and not on every child in the cohort. As per WHO and the Global IDF/ISPAD guidelines, autoantibody testing is not a criterion for diagnosis of type 1 diabetes in children. However, autoantibody tests were sometimes performed in children who were obese or above the age of 10 years, had a family history of type 2 diabetes in first degree relatives, and/or exhibit skin manifestation of insulin resistance. Monogenic diabetes is often diagnosed post puberty. Neonatal diabetes due to glucokinase deficiency or pancreatic aplasia was diagnosed in children of up to six months age; and diabetes secondary to other conditions such as cystic fibrosis-related diabetes and drug induced diabetes related to steroid therapy.

### Statistical analysis

Yearly incidence of DKA was calculated as the percentage of newly diagnosed T1DM patients who presented with DKA in the period between 2011and 2013. Poisson regression was used to test for change in incidence over the three years’ period. Chi-square testing was used to compare differences in the frequency of DKA found in our sample (2011–2013) to the frequency previously reported in the literature (1992–1997)^8^. A multivariable-adjusted logistic regression model was applied to identify factors (including age at diagnosis, gender, and diabetes history of first degree relatives) that are potentially associated with DKA. An unadjusted odds of DKA associated with each of the presented clinical features, were also assessed in separate bivariate logistic regression model. Among subjects who had DKA at diagnosis, ordinal logistic regression was carried out to assess whether any of the above-mentioned variables were associated with increasing DKA severity.

### Ethical aspects

The adopted procedures for data analysis conformed to Helsinki Ethical principles^20^. The Ethical Review Committee at Dasman Diabetes Institute approved the study.

## Results

**Figure 1:**
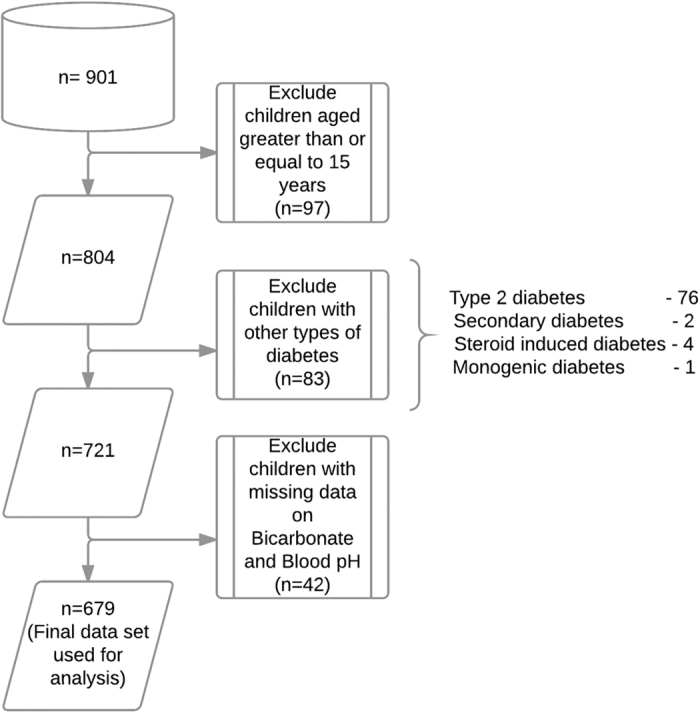
Flowchart demonstrating the exclusion criteria used to derive the final data set on children presented with DKA at the onset of T1DM.

A total of 901 children were registered and 679 of these children passed through the exclusion criteria (Fig. 1). 76 children were diagnosed with type 2 diabetes, 2 with secondary diabetes, 4 with steroid induced diabetes, and 1 with monogenic diabetes were excluded. The majority of patients in the dataset were Kuwaiti children (488/679; 71.9%) and 191 (28.1%) were non-Kuwaitis resident in Kuwait. Non-Kuwaiti Arabs constituted (22.8%), Asians (3.8%), Africans (0.6%) and Caucasian (0.9%) of the total number of children. Of the 243 children in whom pancreatic autoantibody tests were performed, 185 (76.1%) were GADA positive with the remaining 58 (23.9%) children GADA negative. The 58 children with negative GADA results were assigned to the diagnosis of type 1b diabetes.

The proportion of DKA (pH < 7.3) among the total cohort was 33.6% (95% CI: 30.1–37.3). When DKA was further classified into mild (pH < 7.3) and severe DKA pH < 7.1) the proportions were 24.8% and 8.8% respectively.

Basic statistics on patients with and without DKA are summarized in Table 1. The age range of these children was 0.7–14.9 years and the majority (42.7%) was aged between 5–9 years. Although girls presented more frequently (127/356; 35.7% of girls) with DKA than did boys (101/323; 31.3% of boys), the difference was not statistically significant (p = 0.3). The mean age at diagnosis of T1DM was seen as 8.2 ± 3.5 for the whole cohort, and 8.5 ± 3.6 and 7.9 ± 3.4 for boys and girls respectively. No difference was seen between patients with or without DKA (8.1 years vs. 8.2 years; p = 0.7). HbA1c and random plasma glucose levels were seen higher among DKA patients than among non-DKA patients - (11.9% vs. 10.9%; p < 0.001) and (27.5 vs. 24.0; p < 0.001), respectively.

It is known that younger age at diagnosis of DKA is associated with lower HbA1c but higher glucose concentrations^21^,^22^. We too found a similar observation with the cohort from Kuwait. The mean value for HbA1c in children less than 2 years (n = 28) was lower than that in the higher age group (9.39 ± 1.69 versus 11.3+2.42, p  < 0.001) and the mean value for random blood glucose in the age group of less than 2 years was higher than that for the age group (29.50 ± 6.94 versus 25.01+8.78, p  < 0.01).

**Figure 2:**
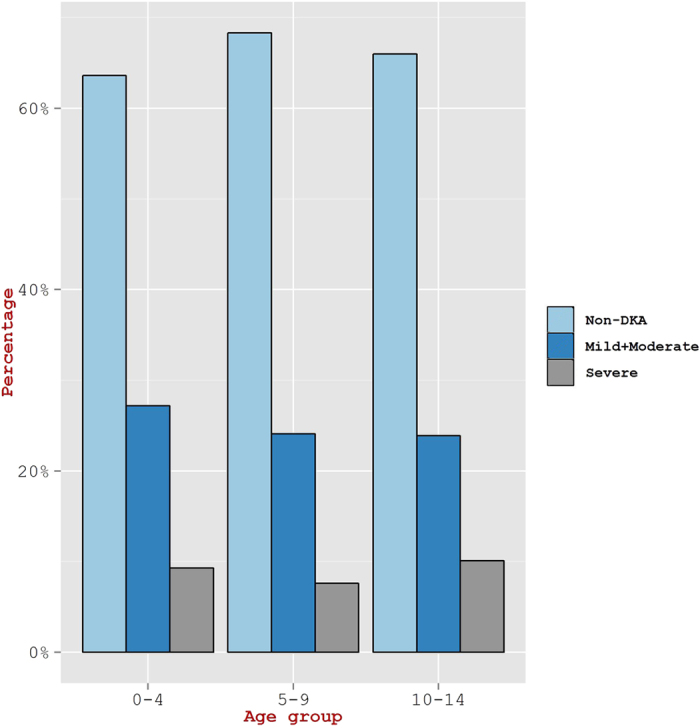
Extent of DKA severity in children from three different age groups.

**Figure 3:**
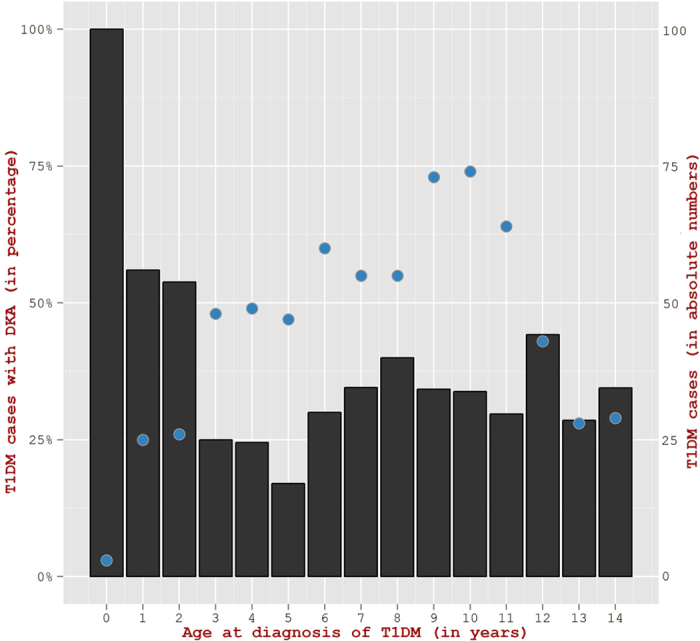
Percentage of T1DM children with DKA according to age at diagnosis of T1DM (Black bars on left Y-axis). Total number of new T1DM children in Kuwait from 2011–2013 (Blue circles on right Y-axis).

**Figure 4:**
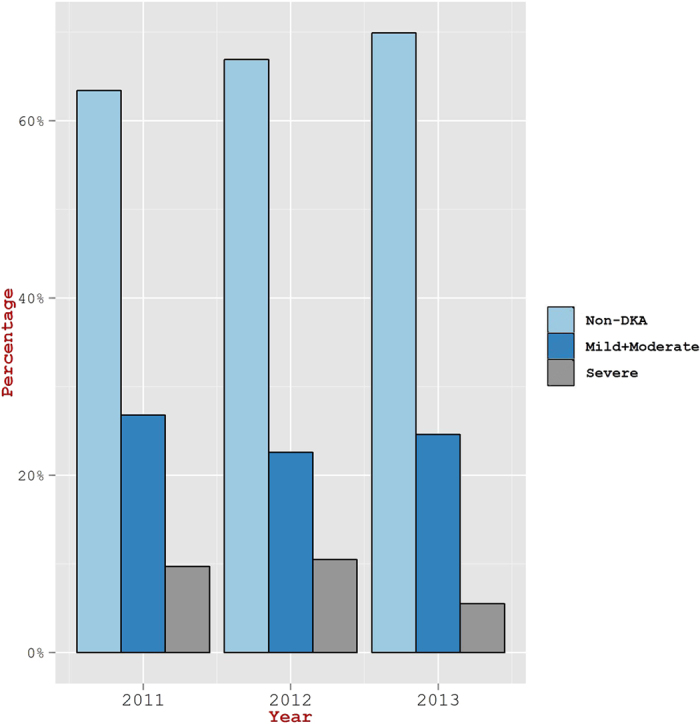
Observed year-wise extent of DKA Severity in children diagnosed for T1DM during the years 2011 to 2013.

Although, the frequency for incidence of DKA was seen similar in all age groups of 0–4 years, 5–9, and 10–14 (Fig. 2), children in the age group of ≤2 years exhibited a higher DKA incidence rate as compared to the age group of 2–14 years (60.7% versus 32.4%, p  <  0.05) (Fig. 3). In a similar manner, the percentage of children in ≤2 years age group having severe DKA was seen higher than in the age group of 2–14 years (21.4% vs. 8.3%; p  <  0.05). The yearly proportion of patients with DKA varied from 36.6% in 2011 to 30.1% in 2013 (Fig. 4). However, the overall change in the incidence of DKA over the three year period was not statistically significant (p = 0.4). Also, the proportion of children with severe DKA did not change over the study period (p = 0.16), despite marked variation from year to year.

17.0% of the children had a history of T1DM in first degree relatives. Analysis for association between family history of diabetes and onset of DKA indicates that family history of T1DM with any of the first degree relatives showed a protective effect on DKA (OR = 0.44; 95% CI = 0.27–0.71). We further found that gender was not associated with DKA frequency (OR = 0.82; 95%CI = 0.59–1.13). Clinical features associated with DKA patients are listed in Table 2. Symptoms showed association with DKA - dehydration (OR = 11.23 CI = 7.27–17.37), vomiting (OR = 7.02 CI = 4.42–11.13) and abdominal pain (OR = 2.45; 95%CI = 1.73–3.49); nocturnal enuresis did not show significant association with DKA (OR = 0.75; 95%CI = 0.54–1.04). Ordinal logistic regression models for the three severity categories of DKA revealed that higher odds of patients with more severe DKA are in the groups of patients that show clinical features like vomiting (OR = 2.41; 95% CI = 1.37–4.25) and dehydration (OR = 1.75; 95% CI = 1.04–2.95). Onset age, gender and family history of T1DM showed no significant association with severity levels.

Significant differences were found in incidence of DKA between Kuwaiti and non- Kuwaiti children (31.1% vs. 39.8%; p  < 0.05) (Table 1) and two health regions had a higher incidence of DKA (46.8 and 43.8%) while it ranged between 25.3 to 35.0% in the other areas. These areas are less urbanized and more disadvantaged than other areas in Kuwait.

There were no deaths registered in this cohort. Six children presented in coma and two developed cerebral edema in the course of treatment. Another child developed cerebral thrombosis but recovered completely. No significant morbidity was present on follow up.

Compared to the earlier study from Kuwait in the 1990s^9^, which employed standardized data collection as per the DiaMond protocol and carried out by the same investigators, the recent incidence data are significantly reduced, from 49% to 33.6% in 2011–2013 (p < 0.001).

## Discussion

Reports of DKA at presentation of T1DM from the Arab countries are scarce and are based on small sized data sets^4^. In this study, we present results based on our collection of data on 679 children (0–14 years) from Kuwait.

The incidence of T1DM has doubled in Kuwait over the last two decades, with the recent incidence rate of 40.9% (95% CI 37.4–44.6) per 100000 per year in Kuwaiti children aged 0–14 years (unpublished data). In this study, mild/moderate DKA at diagnosis of T1DM was seen present in 24.8% of children aged 0–14 years while severe DKA was seen in 8.8% of cases. An inverse correlation between the frequency of diabetic ketoacidosis and the background incidence rate was found in Europe^23^ and accordingly the incidence of DKA was expected to be low in Kuwait. However, there is conflicting evidence on the associated effects on DKA rates^24^,^25^,^26^.

Previous published reports on the incidence of DKA at onset of T1DM in Kuwait in the 1990s indicated that nearly half of the children (49%) in children aged 0–14 presented in DKA and in 23.5% it was severe^9^. More recently in the period between 2000 and 2006^11^ 37.7% of children aged 0–12 years presented with DKA of varying severity. Compared to the earlier study from Kuwait^9^, which employed similar standardized data collection as per the DiaMond protocol, the recent incidence data are significantly reduced, from 49% to 33.6% in 2011–2013 (p < 0.001).

Data from 24 centers in Europe, the EURODIAB study, demonstrated large variations in the incidence of DKA at diagnosis in children across Europe, and a frequency of 33% and 9% as mild and severe DKA was reported respectively^23^. In our study, the frequency of severe DKA (8.8%) is comparable and significantly lower than in Spain (17.8%)^27^ or France (14.8%)^28^, but was much higher than that reported from Denmark (1.7%)^29^.

In the present study, the frequency of DKA was notably higher in young children under two, 60.7% vs 32.4% in older children. The Pediatric Diabetes Consortium in the US reported similar results where the incidence of DKA was 34% and 54% in children aged 0–14 and <3 years respectively^30^. Also in Northern Finland, the frequency of DKA remained high during 1992–2001, and 39.1% of children aged <2 years developed DKA at diagnosis, despite a high incidence of T1DM in this country^31^. It is seen that not only the incidence of ketoacidosis was high in children less than 2 years of age but also the proportion of children from this age group with severe ketoacidosis is high. We have also seen lower HbA1c and higher glucose levels in children less than 2 years. It is probably the case that in very young children of less than 2 years, a more rapid loss of beta cell function occurs within a shorter duration of symptoms^32^ (as compared to children of 2–14 years) resulting in higher DKA rates, and higher blood glucose levels in children less than 2 years. As HbA1c represents the average blood glucose levels in the past three months, the shorter duration of symptoms in children less than 2 years compared to children of 2–14 years argues for lower HbA1c levels.

The two governorates with the highest incidence of DKA greater than 40%, represent communities with a large proportion of low income families, many of whom are non- Kuwaitis, and awareness of DKA and its symptoms might be limited within the population. In one area in particular, women were disadvantaged in comparison to their counterparts in the more urbanized areas with respect to educational level and employment^33^ Furthermore, non Kuwaitis residents in Kuwait had a significantly higher incidence of DKA (39.8 vs 31.1% p  <  0.05) at the onset of T1DM, and although all residents (Kuwaiti and non-Kuwaiti) have access to state sponsored health care, differences in health care seeking behavior between different socio-economic groups can be an important factor when interpreting our results. Health seeking behavior of non- Kuwaitis is not well understood but factors such as language barriers and lack of information about health services can be assumed to contribute to less efficient use of resources. Furthermore, the health hazards associated with expatriates are more severe and the prevalence of type 1 diabetes (2.8% versus 2.3%), type 2 diabetes (33.3% versus 25.4%), and hypertension (37% versus 28%) is higher in expatriates than nationals^34^. Consistent with other reports^4^, the family history of T1DM (and T2DM) in any of the first degree relatives showed a protective effect on the incidence of DKA (OR = 0.46; 95% CI = 0.28–0.73).

Worldwide, the prevalence of mortality from DKA occurs in 0.3–1% and is principally due to cerebral edema^35^. No deaths were reported in our cohort of children and it is unlikely that cases have been missed as the symptoms and signs of DKA are dramatic and accessibility of healthcare in the country is ensured to all citizens. It has been suggested that a large proportion of the variation in DKA is due differences in a country’s economic position and healthcare provision, with higher frequency of DKA in countries with lower GDP and hence lower expenditure on healthcare^36^. Kuwait is a rich country with high GDP^37^, high literacy and low infant mortality rates^38^. In 2013, the health expenditure per person with diabetes was $ 1,886, which compares favorably with other countries in the region^39^.

DKA continues to occur at substantial rates worldwide, yet there are opportunities to prevent DKA through public awareness campaigns on early symptoms in order to encourage early diagnosis and detection of metabolic deterioration before DKA develops^39^. This has been demonstrated in countries both from the region as in the case of Saudi Arabia as well as elsewhere (as in Parma). For example, a study from Saudi Arabia^5^ indicated that following awareness campaigns, the DKA rate dropped from 48% in 2010 to 39% in 2014 and 15.8% had severe DKA compared to 26.1% in 2005–2010 (p < 0.01). Studies in Parma Italy^40^,^41^, have shown that it is possible to achieve a marked decrease in the frequency of DKA through educational campaigns that focused on one of the earliest symptom of diabetes namely, nocturnal enuresis in a dry child, and targeted schools and primary healthcare physicians. During the 8 years of the campaign the cumulative frequency of DKA dropped from 78% to 12.5% in 6–10 years’ old^41^, demonstrating great success. In 2013 we have launched an awareness campaign similar to the one implemented in Parma, Italy, and we have been monitoring the results over the last 3 years. We hope this will further reduce the frequency of DKA at presentation in Kuwait – the results from Saudi Arabia^5^ illustrates that this is possible in the region.

Risk factors for presentation with DKA at diagnosis of T1DM include younger age at onset of T1DM, ethnicity, and subscription to healthcare private insurance^4^,^42^,^43^. Furthermore, low socioeconomic status and low parental education have been found associated with increased risk of DKA^43^,^44^. Type 1 diabetes is a major risk factor for diabetic ketoacidosis across the world and is prevalent in Kuwaiti children^45^. Infection, particularly urinary tract infection (UTI), is known as the most common precipitating cause for DKA. UTI has been shown to be common in infants and children in Kuwait^46^. Pneumonia account for the majority of infections that act as risk factors for DKA. Up to 40% of clinical infections are caused by a pneumococcal strain that is resistant to at least one drug, and 15% are due to strains resistant to three or more drugs^47^. The prevalence of penicillin-resistant S.pneumoniae (PRSP) in the pediatric and elderly populations exceeds 60%; almost half of these strains are multidrug resistant^48^,^49^.

The strength of our study is that it describes the trends of DKA at onset of T1DM in a large cohort from Kuwait, which has the highest incidence of T1DM in the region. The region has been underrepresented in studies reported in the literature. Data were collected based on a prospective national population based study, which captured information routinely recorded at initial presentation, and covers both Kuwaiti children and expatriates. Hence the study covers aspects relating to different ethnicities.

There are a number of limitations to this study. First, we have not collected information on family income and parental education; secondly the study lacks information on duration of symptoms or degree of weight loss at presentation. A third limitation is that data was not recorded on the number of visits to the emergency room or primary health care centers before presentation to assess the causes of delay in diagnosis.

## Conclusions

Over the last two decades, we have witnessed a dramatic increase in the incidence rates of T1DM. Although, we observed a decline in the incidence of DKA in Kuwait, it is still high, particularly in young children. The severity of DKA, on the other hand, has decreased in comparison to previous reports from Kuwait and are consistent with data reported from developed countries.

An awareness campaign is needed to increase public awareness among health care providers, parents and school teachers in Kuwait.

## Additional Information

**How to cite this article**: Shaltout, A. *et al*. Ketoacidosis at first presentation of type 1 diabetes mellitus among children : a study from Kuwait. *Sci. Rep.* **6**, 27519; doi: 10.1038/srep27519 (2016).

## Supplementary Material

Supplementary Information

## Figures and Tables

**Table 1:** Characteristics of 679 children and adolescents (0–14 years) with T1DM.

**Characteristic**		**DKA frequency**		**p value**
		**n/N**	**(%)**	
All	0–14	228/679	33.6	
Age at diagnosis (years)				p = 0.6
	0–4	55/151	36.4	
	5–9	92/290	31.7	
	10–14	81/238	34.0	
Year				p = 0.4
	2011	94/257	36.6	
	2012	79/239	33.1	
	2013	55/183	30.1	
Sex				p = 0.3
	Boys	101/323	31.3	
	Girls	127/356	35.7	
Ethnicity				p < 0.05
	Kuwaiti	152/488	31.1	
	Non- Kuwaiti	76/191	39.8	
Family history of diabetes				p < 0.001
	Yes	24/119	20.2	
	No	204/560	36.4	
Heath Region				p < 0.05
	Ahmadi	44/174	25.3	
	Capital	38/126	30.2	
	Farwaniya	39/112	34.8	
	Jahra	29/62	46.8	
	Hawalli	49/140	35.0	
	Mubarak alKabeer	28/64	43.8	

**Table 2:** Clinical features associated with DKA in Kuwait 2011–2013.

**Symptoms**	Percentage of children inDKA with symptom (n)	Percentage of children inNon-DKA with symptom (n)	**p-value**
Cerebral edema	1 (2)	0 (0)	0.214
Blurring of vision	1 (2)	0 (0)	0.214
Coma/altered consciousness	3 (6)	0 (1)	0.01
Acanthosis nigricans	0 (1)	2 (7)	0.371
Dizziness	4 (8)	0 (2)	0.005
Obesity	2 (4)	2 (7)	1
Skin infection	2 (4)	2 (11)	0.76
Vulvovaginitis	6 (14)	4 (19)	0.36
Vomiting	33 (76)	7 (30)	<0.001
Dehydration	48 (109)	8 (34)	<0.001
Acidosis	52 (119)	7 (30)	<0.001
Abdominal pain	39 (88)	20 (92)	<0.001
Nocturnal enuresis	36 (83)	43 (195)	0.10
Loss of weight	79 (181)	72 (326)	0.064
Polydipsia	87 (199)	91 (409)	0.22
Polyuria	92 (209)	96 (433)	0.03
