# Supplementary material for: Ketoacidosis at first presentation of type 1 diabetes mellitus among children: a study from Kuwait
Source: Sci Rep. 2016 Jun 22;6:27519. doi: 10.1038/srep27519 (PMC4916451; doi:10.1038/srep27519)
Supplement: Supplementary Information [file srep27519-s1.docx]

# Supplementary information file

# Title: Ketoacidosis at first presentation of type 1 diabetes mellitus among children and adolescents: a study from Kuwait.

Azza Aly Shaltout^1*^, Arshad Mohammed Channanath^2^ , Thangavel Alphonse Thanaraj^2^ , Dina Omar^1^, Majedah Abdulrasoul^3^, Nabila Zanati^4^, Maria Almahdi^4^, Hessa Alkandari^5^ Dalia Al-Abdulrazzaq^3^ Linda d’Mello ^1^, Fawziya Manadani ^6^ Ayed Alanezi ^7^ Eman AlBasiry^8^ and M Alkhawari ^9^

**Consortia:** The following persons and institutions participated in CODeR.

The Steering Group for the Study of Childhood Diabetes. Ministry of Health ^10^: Rehab Al Wotayan; Primary Health Care Centers ^11^ : Zekrayat Al Shemmari, Huda Ghareeb, Alfred Michael, Wafik Saba , Sara Al Jassar, Yasmine Khuraibit; Kuwait Oil Company Hospital, Ahmadi ^12^ : Hosam Sorrour; Department of Planning and Healthcare Development ^13^: Dalia Badawi ; IT Department, Dasman Diabetes Institute ^14^: Yousuf Almeheymi, Ehab Haroon.

Amiri Hospital ^9^: Mariam Qabazard, Hala Alsane; Al Jahra Hospital^7^: Hossam Mahmoud; Mubarak Hospital ^8^ : Faisal Al Shawaf; Sabah Hospital ^6^: Nada Al Terkait , Afaf Al Adsani, Esraa Maarafi; Farwaniya Hospital ^5^: Abeer Al Tararwa:
